# Nonmotor-Related Quality of Life in Parkinson's Patients with Subjective Memory Complaints: Comparison with PDQ-39

**DOI:** 10.1155/2020/7953032

**Published:** 2020-04-20

**Authors:** Alexis Lionel Jenny, Antonia Meyer, Ivana Handabaka, Pasquale Calabrese, Peter Fuhr, Ute Gschwandtner

**Affiliations:** ^1^Department of Neurology, Hospital of the University of Basel, Petersgraben 4, Basel 4031, Switzerland; ^2^Division of Molecular and Cognitive Neuroscience, Neuropsychology and Behavioural Neurology Unit, University of Basel, Basel, Switzerland

## Abstract

**Background:**

Parkinson's disease (PD) is associated with cognitive decline, progressing from subjective memory complaints (SMC) via mild cognitive impairment (MCI) to dementia. SMC are only measurable by an interview and thus rely on individuals reporting a subjectively perceived worsening of cognitive functioning. Cognitive decline is accompanied by a reduction in quality of life (QoL); however, the extent to which SMC manifest a reduction of QoL remains unclear.

**Objective:**

To determine the association between SMC and deterioration of QoL in patients suffering from PD.

**Methods:**

A total of 46 cognitively unimpaired PD patients (29 men and 17 women) completed PDQ-39, two assessments to measure SMC (Informant Questionnaire on Cognitive Decline in the Elderly (IQCODE) and a Self-Assessment questionnaire), Beck Depression Inventory (BDI), and Beck Anxiety Inventory (BAI). Multiple regression modelling was conducted to investigate the confounding effect of depression and anxiety.

**Results:**

The PDQ-39 domain cognitions, but not the PDQ-39 sum score, correlated significantly with the SMC Self-Assessment questionnaire (*r* = 0.57; *p* < 0.001). The conducted regression model indicates a significant confounding effect of depression and anxiety (*p* < 0.001, *R*^2^ = 0.55).

**Conclusion:**

In our study, SMC is significantly related to a reduction of cognitive QoL. In addition, we observed significant relation to anxiety and depression levels. In contrast to our main hypothesis, we found no association with overall QoL; this lack of association could be due to unstandardized questionnaires and emphasizes the need of validated tools for evaluating SMC.

## 1. Introduction

Parkinson's disease (PD) is a neurodegenerative disease characterized by a progressive course of both motor and nonmotor symptoms. It has been shown that patients suffering from PD may experience pronounced decrease in cognitive function leading to a decrease of their quality of life (QoL) [1].

One of the first signs of cognitive decline in PD patients is characterized by subjective memory complaints (SMC), which may act as a precursor announcing mild cognitive impairment (MCI) and dementia, respectively [2]. In contrast to MCI, SMC are not objectively quantifiable through a standardized neuropsychological test, but only based on reports by the patients and their caregivers [3]. A review of several studies states that the prevalence of SMC in PD patients varies between 25% and 50%. Higher age, female sex, and lower education have been shown to put patients at higher risk for the further development of SMC [4].

Recent studies have found that SMC are primarily related to neuropsychiatric syndromes such as depression and anxiety [5–7].

In line with many other chronic neurological diseases, PD has a negative effect on patients' QoL, where psychological symptoms such as depression and cognitive impairment are considered major determinants of deterioration in QoL. For this reason, a greater value is placed on appropriately measuring QoL and bringing it into the context of the nonmotor-related aspects of the disease [8].

The aim of this study was to investigate the extent to which SMC impact PD patient's QoL (assessed by the PDQ-39). We wanted to evaluate whether a deterioration of QoL could be found in PD patients with SMC prior to measurable cognitive impairment.

We examined the three following main hypotheses:

1. A significant negative association between SMC and QoL in patients with PD exists.
2. A significant correlation between SMC and the PDQ-39 domains emotional well-being, cognitions, and communication can be established.
3. Anxiety and depression are confounding factors affecting SMC and QoL.

## 2. Methods

### 2.1. Sample

Eighty six patients diagnosed with idiopathic PD according to the UK Parkinson's Disease Brain Bank Criteria were recruited [9]. Patients were recruited from the Movement Disorders Clinic at the University Hospital Basel, or were enlisted via advertisements or through assignment by other clinicians. Exclusion criteria were the presence of moderate or severe dementia (DMS-IV, MMS <24), psychosis, other brain diseases, secondary Parkinson's disease, or insufficient knowledge of the German language. For the present study, 15 PD patients were excluded from the analysis due to missing data of PDQ-39. Another 25 patients in whom MCI could be diagnosed according to the level II criteria of Litvan et al. [10] were also excluded. The total group of 46 (29 men and 17 women) consisted of cognitively unimpaired PD patients. The characteristics of the sample is shown in Table 1.

### 2.2. Questionnaires

To investigate QoL, the Parkinson's Disease Questionnaire (PDQ-39) was used [11]. The PDQ-39 consists of 39 questions distributed across eight domains, which are summed up to a total score. These eight domains are mobility, activities of daily living, emotional well-being, stigma, social support, cognitions, communication, and bodily discomfort [12]. Each item is rated on a five-point scale: 0 = never, 1 = rarely, 2 = sometimes, 3 = frequently, and 4 = always/not possible at all [11]. Each dimension total score ranges from 0 (never have difficulty) to 100 (always have difficulty). Lower scores reflect better QoL. A PDQ-39 sum score (PDSI) is calculated as the mean value of the eight individual domain scores.

SMC was assessed using two questionnaires. First, the Informant Questionnaire on Cognitive Decline in the Elderly (IQCODE), which is completed by a relative or caregiver. It is used to detect cognitive change of the patient over the course of the last two years [13]. The questionnaire consists of 16 items. Each item is rated on a five-point scale from 1 (much improved), 2 (a bit improved), 3 (not much change), and 4 (a bit worse) to 5 (much worse). The final IQCODE score is calculated as the mean of all items. The cutoff value is 3.19, and higher values indicate severer cognitive change [14, 15]. In our study, we assumed SMC to be present if a relative/caregiver reported an IQCODE value greater than 3.19 but no neuropsychological deficits were measureable.

As a second measure for SMC, the Self-Assessment questionnaire, which was developed by the Memory Clinic Basel, was conducted. Patients are asked to indicate whether they have difficulties in any of the following seven domains: remembering dates and appointments, keeping track of spatial orientation, difficulties in dealing with reading and TV or radio, recognizing connections, finding words, keeping the names of acquaintances, and organizing invitations or excursions. In our study, we assumed SMC to be present if patients claimed to have difficulties in at least one domain, but no objective cognitive impairment was measured.

### 2.3. Further Measurements

To be able to exclude PD patients with objective cognitive impairment (MCI), a comprehensive neuropsychological test battery, and Level II diagnostic criteria of the Movement Disorder Society Task Force guidelines for the diagnosis of PD-MCI [10] were applied.

The Unified Parkinson's Disease Rating Scale (UPDRS) was completed by an experienced neurologist to evaluate the severity of motor impairment and other impairments related to PD. A maximum of 199 points are achievable, where a score of 199 points implies a high and 0 points implies no disability [16]. The Levodopa Equivalent Dose (LED), was calculated according to Tomlinson's study [17]. The patients additionally completed the Beck Depression Inventory (BDI) and the Beck Anxiety Inventory (BAI). A standardized questionnaire to ascertain the patients' educational status was applied. It included questions about their schooling, vocational training, further education, and positioning in the working field. The measurement leads to a score between 0 (lowest) and 20 (highest).

### 2.4. Statistical Analysis

To test the relation between the two SMC assessments and the PDSI, a series of linear regression models were calculated. In the next step, we calculated the correlation coefficient between PDQ-39 domains and SMC using Spearman's rank correlation coefficient. To investigate a confounding effect of depression and anxiety on the PDSI score, a nested generalized model was conducted.

## 3. Results

The first hypothesis that SMC is related to the quality of life of PD patients is rejected. Neither IQCODE nor the SMC Self-Assessment questionnaire correlated with PDSI (Table 2).

The second aim was to investigate whether there is an association between SMC and the three domains of the PDQ-39, namely, emotional well-being, cognitions, and communication. The PDQ-39 domain cognitions correlated significantly with the SMC Self-Assessment questionnaire (*p* < 0.001, *r* = 0.572). For the IQCODE, we observed no significant correlation with any of the three domain scores (Table 3).

The third hypothesis investigated whether anxiety and/or depression are confounding factors of SMC and PDSI. Both BAI and BDI showed a significant association with PDSI (Table 4). There is a significant correlation between SMC self-assessment and BDI (*p*=0.008, *r* = 0.388). Adding SMC assessment did not increase the explained variance of QoL, suggesting no significant additional impact of the SMC on QoL beyond anxiety and depression scores (Tables 5 and 6).

## 4. Discussion

The aim of this study was to investigate the association between SMC and QoL in cognitively unimpaired PD patients. In the analysis of PDQ-39 domains, we observed a significant correlation of the domain cognitions with the SMC Self-Assessment questionnaire. In the SMC Self-Assessment questionnaire, questions broadly address cognitive abilities (for example, remembering dates and appointments, recognizing connections, or finding words), whereas in PDQ-39 very similar questions are asked within the domain cognitions (for example, how many times in the last month have you had problems concentrating because of your Parkinson's disease? or How many times in the last month did you feel that you had a bad memory because of your Parkinson's disease?). Although in the PDQ-39 the impact cognition on QoL is stressed, we can assume that the two measures meaningfully overlap.

In our study, SMC is related to depression. We could speculate that depression may result in subtle changes of cognitive capacity which in turn reduces QoL in PD patients.

In line with this assumption, the conducted hierarchical linear model showed the best fit between QoL and the indicators for anxiety and depression (BAI and BDI). A significant association between anxiety/depression and QoL has already been found previously [8]. When adding further predictor variables to the model (i.e., the IQCODE or SMC), the explained variance of QoL was not significantly increased (Table 5).

The following limitations must be addressed: first, our sample consists of heterogeneous patients in terms of disease duration and the anxiety score. Furthermore, the investigated sample of PD patients suffered only from slight depression and anxiety.

Second, no significant association between SMC and QoL was found in our study. Our results may be due to methodological problems associated with the assessments of SMC. The validity of the SMC Self-Assessment questionnaire used in this study is not proven and serves mainly as a rough assessment cognitive functioning. Furthermore, we observed no association between the two measurements. On the one hand, this could be due to inaccurate information from relatives, and on the other hand, statements by relatives and patients regarding SMC often contradict each other. Studies in related fields reported substantial discrepancies between statements of patients and their caregivers [18, 19].

In conclusion, our study did not find a significant correlation between SMC and total QoL. However, we found SMC to be significantly related to a reduction of cognitive QoL. However, in SMC, the symptoms may be more subtle and difficult to capture by reports of affected patients and their relatives.

## Figures and Tables

**Table 1:** Demographic data of subjects.

Sample	46 (17 women, 29 men)	
	Median	Range
Age (years)	67	45–82
Education status (0–20)	14	9–20
BDI score	6	1–15
BAI score	8	0–42
PDSI (0–100)	29	2–66
PDQ-39 emotional well-being	14.6	0–75
PDQ-39 cognitions	25	0–56.5
PDQ-39 communication	8.3	0–66.7
SMC self-assessment score (0–6)	1.5	0–6
IQCODE score (0–5)	3.063	2.938–3.688
UPDRS (0–199)	15	0–36
LED (mg)	537	0–2129
Disease duration (years)	2	0–19

Abbreviations and scores are explained in the above sections.

**Table 2:** Generalized linear model coefficients for PDSI with both SMC assessments.

							95% CI	
Model		Unstandardized	SE	Standardized	t	p	Lower	Upper
1	Intercept	−35.557	43.080		−0.825	0.414	−122.435	51.321
	IQCODE	19.962	13.786	0.209	1.448	0.155	−7.841	47.764
	SMC self-assessment	2.523	1.476	0.247	1.709	0.095	−0.454	5.500

**Table 3:** Spearman correlations (pairwise) of three selected PDQ-39 domains with both SMC assessments.

			Spearman (rho)	*p*
IQCODE	SMC self-assessment	0.063		0.678
IQCODE	Emotional well-being	−0.015		0.922
IQCODE	Cognitions	0.119		0.430
IQCODE	Communication	0.221		0.139
SMC self-assessment	Emotional well-being	0.213		0.155
SMC self-assessment	Cognitions	0.572	^*∗∗∗*^	<.001
SMC self-assessment	Communication	0.143		0.345
Emotional well-being	Cognitions	0.292	^*∗*^	0.049
Emotional well-being	Communication	0.361	^*∗*^	0.014
Cognitions	Communication	0.393	^*∗*^^*∗*^	0.007

^*∗*^ *p* < 0.05, ^*∗∗*^*p* < 0.01, and ^*∗∗∗*^*p* < 0.001.

**Table 4:** Spearman's correlation between BDI, BAI, IQCODE, and SMC self-assessment with PDSI.

							95% CI		
Model		Unstandardized	Standard error	Standardized	t	*p*		Lower	Upper
1	Intercept	−38.207	30.666		−1.246	0.220		−100.138	23.724
	BDI	1.230	0.542	0.289	2.272	0.028		0.137	2.324
	BAI	1.141	0.255	0.523	4.468	<.001		0.625	1.656
	IQCODE	15.410	9.781	0.161	1.575	0.123		−4.344	35.163
	SMC self-assessment	0.989	1.150	0.097	0.860	0.395		−1.333	3.312

**Table 5:** Nested generalized linear model summary.

Model	*R*	*R* ^2^	Adjusted *R*^2^	RMSE	*R* ^2^ change	F change	df1	df2	*p*
0	0.743	0.552	0.531	11.602	0.552	26.483	2	43	<.001
1	0.751	0.564	0.533	11.58	0.012	1.166	1	42	0.286
2	0.763	0.581	0.552	11.346	0.03	2.967	1	42	0.092

Model 0 includes BDI and BAI; Model 1 contains Model 0 plus SMC self-assessment; Model 2 contains Model 0 plus IQCODE. PDSI is the dependent variable.

**Table 6:** ANOVA summary.

Model		SS	df	MS	F	*p*
0	Regression	7129.785	2	3564.892	26.483	<.001
	Residual	5788.324	43	134.612		
	Total	12918.109	45			
1	Regression	7286.145	3	2428.715	18.112	<.001
	Residual	5631.963	42	134.094		
	Total	12918.109	45			
2	Regression	7511.746	3	2503.915	19.452	<.001
	Residual	5406.363	42	128.723		
	Total	12918.109	45			

Model 0 includes BDI and BAI; Model 1 contains Model 0 plus SMC self-assessment; Model 2 contains Model 0 plus IQCODE. PDSI is the dependent variable.

## Data Availability

The Excel files used to support the findings of this study are available from the corresponding author upon request.
